# Supplementary material for: Structure of the human RAD17–RFC clamp loader and 9–1–1 checkpoint clamp bound to a dsDNA–ssDNA junction
Source: Nucleic Acids Res. 2022 Jul 12;50(14):8279–89. doi: 10.1093/nar/gkac588 (PMC9371934; doi:10.1093/nar/gkac588)
Supplement: gkac588_Supplemental_File [file gkac588_supplemental_file.pdf]

SUPPLEMENTARY FIGURE 1

A

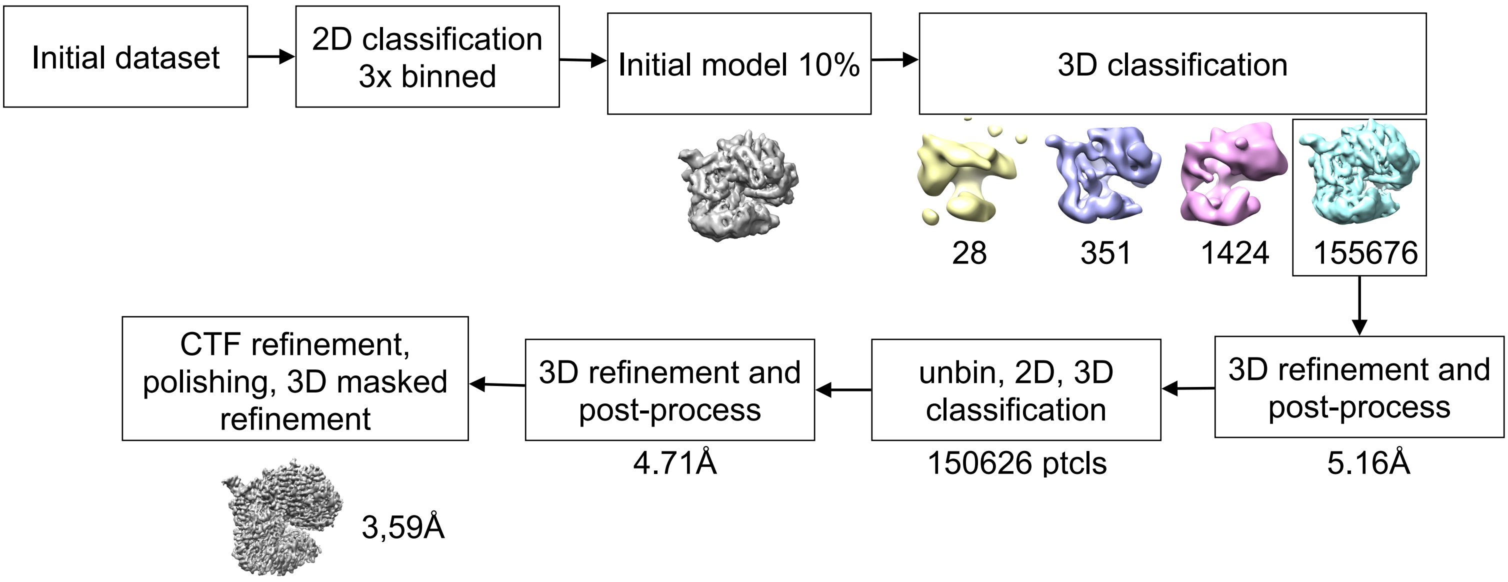

Flow diagram of data processing pipeline followed to give reported structure. All steps used RELION4.0

B

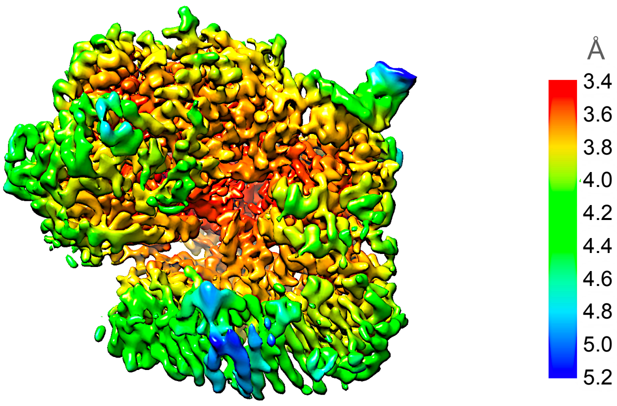

Local variation of resolution in reported structure

C

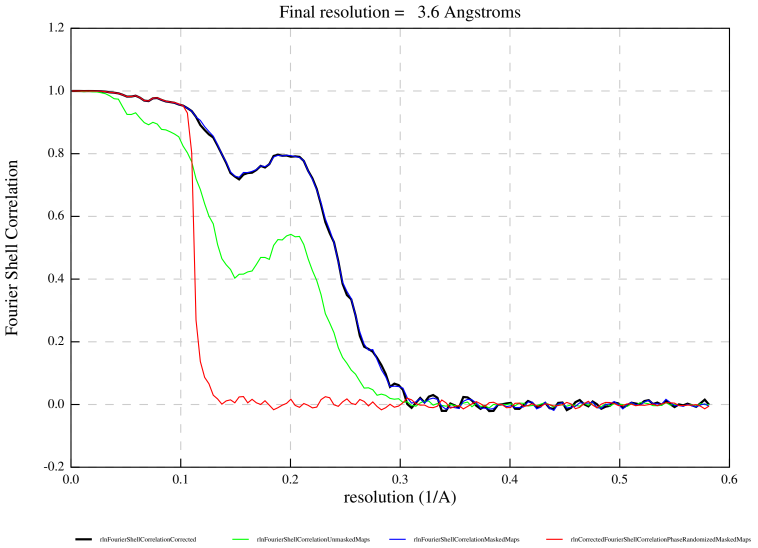

Fourier shell correlation plot for final structure

SUPPLEMENTARY FIGURE 2

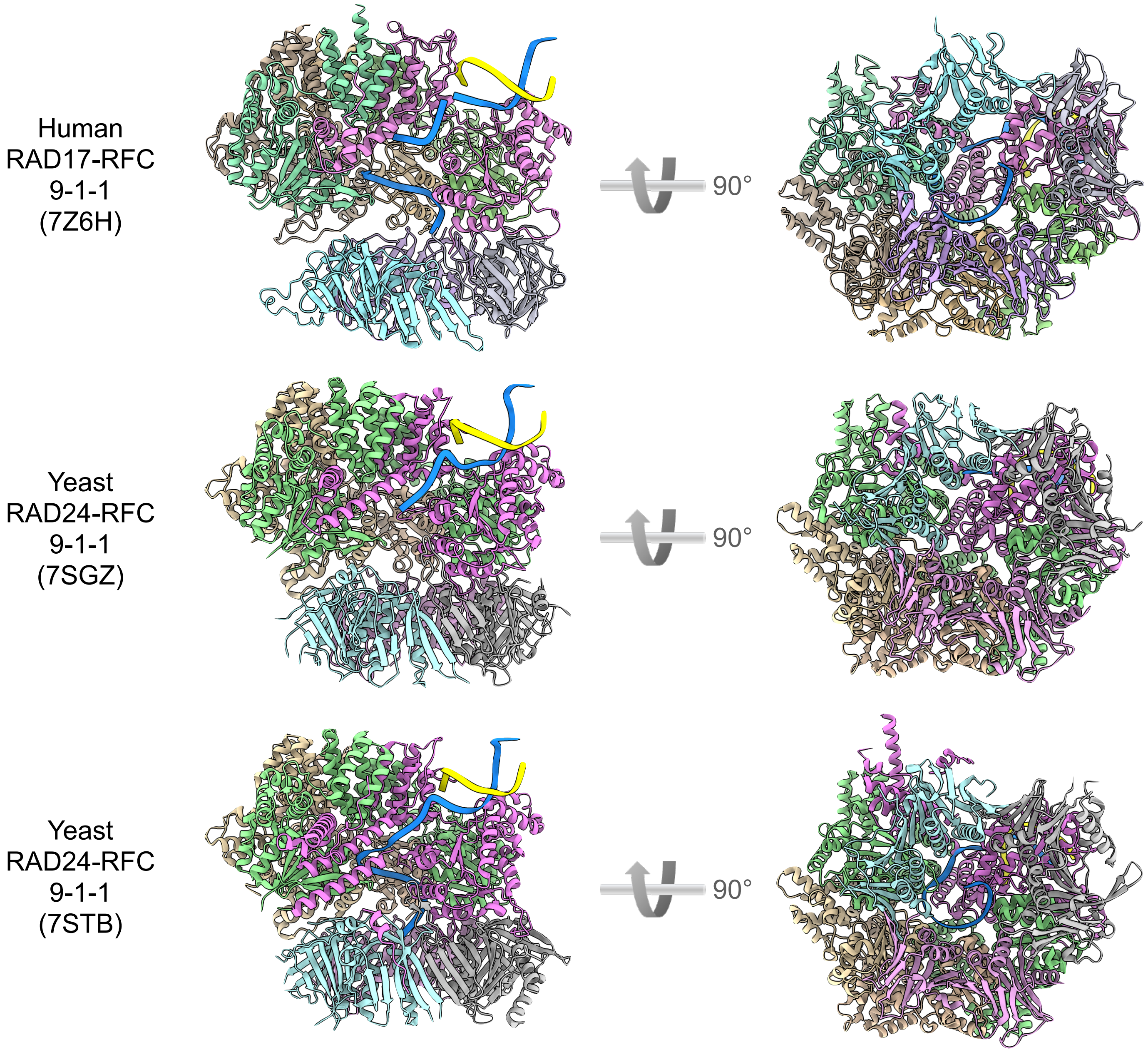

Orthoganol views of superpostion of the closed Human RAD17-RFC 9-1-1 and the two published closed confirmations of the Yeast Rad24-RFC 9-1-1 structures with subunits (or equivalents) coloured as for figure 1

SUPPLEMENTARY FIGURE 3

A

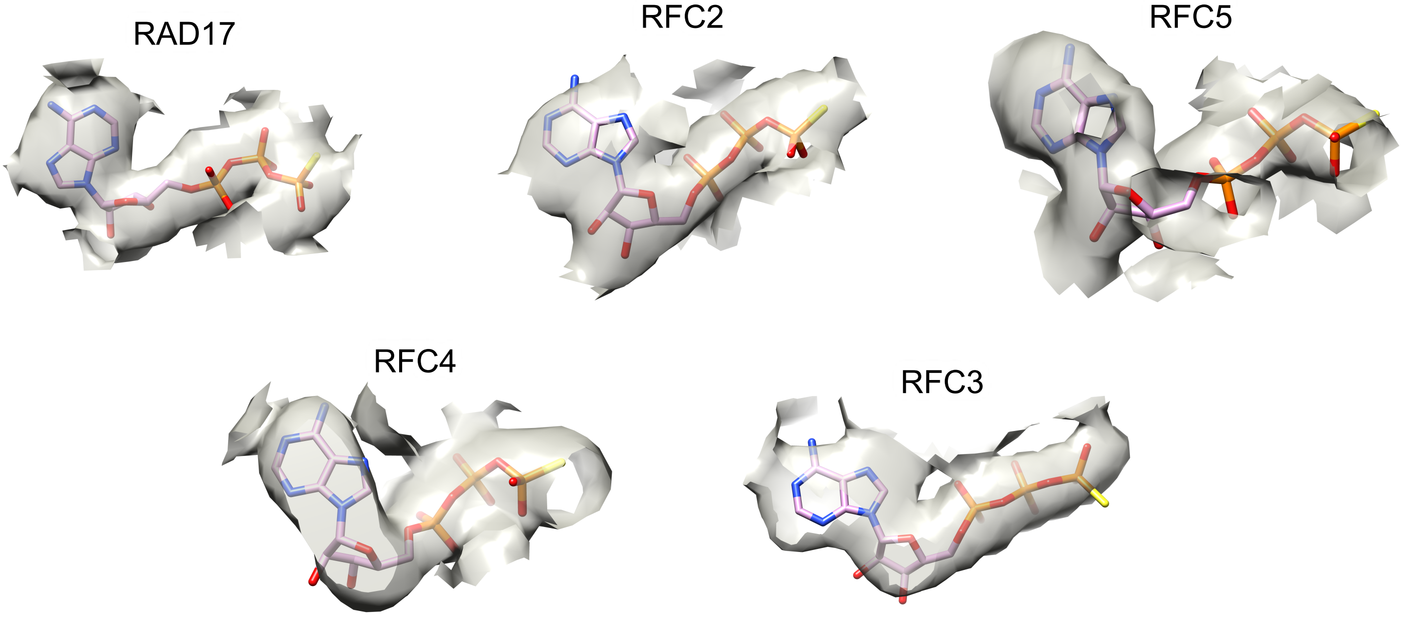

Montage of features in the experimental map corresponding to adenine nucleotides bound to each subunit of the clamp loader. In all cases volumes suggestive of binding of ATPγS are evident.
